# Evaluating counselling skills of community health workers for pregnant adolescents in Limpopo province

**DOI:** 10.4102/sajpsychiatry.v30i0.2217

**Published:** 2024-07-30

**Authors:** Rakgadi G. Malapela, Sheillah H. Mboweni, Patrone R. Risenga

**Affiliations:** 1 Department of Health Studies, School of Social Sciences, University of South Africa, Pretoria, South Africa

**Keywords:** adolescents, community health workers, counselling, evaluation, pregnancy

## Abstract

**Background:**

Adolescent pregnancy carries significant global ramifications. Community health workers (CHWs) play a role in empowering adolescents through counselling skills, thereby promoting informed decision-making for better outcomes.

**Aim:**

The study aims to evaluate the counselling skills of CHWs in their efforts to support pregnant adolescents (PA) in Limpopo province.

**Setting:**

The research was carried out with CHWs in the Mopani and Vhembe districts of the Limpopo province.

**Methods:**

A quantitative descriptive approach was used to evaluate CHWs’ counselling skills for PAs in Limpopo. A sample of 81 respondents was selected using simple random sampling. Ethical approval was obtained. Data were collected using a questionnaire guided by the Theory of Reasoned Action. Descriptive statistics were analysed using Statistical Package for Social Scientists version 24. The questionnaire’s validity was assessed using Cronbach’s alpha, resulting in a correlation coefficient of 0.710.

**Results:**

The study identified significant variations in counselling recommendations. *Preparation*: Using private space with fewer distractions showed considerable variability (standard deviation = 0.218). *Introduction*: High variability was observed in using the SOLER method (standard deviation = 0.316). *Working phase*: Suggesting rather than advising had notable variability (standard deviation = 0.396). *Termination*: Avoiding abrupt endings and informing clients about the session’s conclusion demonstrated variability (standard deviation = 0.283). Additionally, the majority (64%) of the participants found record-keeping unnecessary, which demonstrated the highest variability (standard deviation = 0.482).

**Conclusion:**

The study revealed CHWs’ proficiency in counselling techniques and emphasised the importance of following the counselling stages.

**Contribution:**

The research highlights the importance of evaluating the counselling skills of CHWs and identifying areas for improvement to develop targeted interventions and enhancing health outcomes for PAs.

## Introduction

Adolescence is that distinct period of human growth and life amid childhood and adulthood, between the ages of 10 and 19.^1^ Adolescents are vulnerable to risky behaviours and mental health disorders such as depression and anxiety because of rapid changes in physical, psychological, cognitive and social functioning during this key stage of life.^2^ As a result of risky behaviours such as substance abuse and sexual experimentation, adolescent females are particularly vulnerable to becoming pregnant.

Adolescent pregnancy (AP) continues to be a global public health concern and subsequently has become a priority that needs to be addressed.^3^ The risk of death from complications because of AP is doubled when compared to pregnant women above the age of 20.^4^ Concerns regarding early pregnancy and childbearing before the age of 18, particularly among girls between the ages of 10 and 15, were raised in the 2021 United Nations Children’s Fund (UNICEF) report.^5^ Furthermore, some 16 million adolescents between the ages of 15 and 19 are thought to become pregnant and give birth each year, and in low-and middle-income countries (LMICs), girls under the age of 16 account for 2.5 million of the world’s births or 11.5% of all births. Most of these APs are unwanted and typically occur in rural, underprivileged and disadvantaged areas.^6^ Pregnant adolescents (PAs) are at a higher risk for developing long-term health issues and experiencing societal repercussions when compared to older women.^3^ Approximately 3.9 million unsafe abortions that contribute to 99% of maternal death, and morbidity rates worldwide are some of these health issues. Furthermore, APs expose young girls to societal consequences such as bullying, stigma, intimate partner violence and violence from parents and peers.^3^ These experiences have a lasting impact on the girls’ future, affecting their educational and employment prospects. Consequently, this results in long-term economic dependency, low self-esteem, rejection and may even lead to depression and suicide.^6,7^

The Republic of South Africa’s basic education system promotes access to counselling, care and support for all pregnant learners and those who have young children.^8^ It is through scholarly engagement that communities and higher education can work together to improve the lives of these learners.^9^ Thus, a scholarship community engagement was carried out to provide community health workers (CHWs) with fundamental counselling abilities to help adolescents deal with the effects of and overcome problems associated with pregnancy.

Counselling is a two-way communication process between the client and the trained counsellor to help the client cope with stressors and to make informed decisions.^10^ Counselling does not mean delivering advice and quick fixes but rather empowering teenagers to be aware of their own issues, fears and stressors and to make wise decisions that deal with such challenges.^11^ The World Health Organization (WHO) is urging all stakeholders, including non-governmental organisations (NGOs), to address the challenges faced by teenagers.^3^ This includes encouraging NGOs to develop fundamental counselling abilities to equip teenagers with the necessary tools to navigate the difficulties they encounter. The study conducted in sub-Saharan Africa emphasised the scarcity of studies that address the mental health needs of PAs.^4^ It highlighted the necessity of providing support to help them manage their challenges effectively.

Approximately half of the unintended pregnancies among adolescents aged 15 to 19 result from sexual assault, coerced first-time sexual experiences and difficulties related to accessing or using contraception.^3^ Moreover, these adolescents frequently experience feelings of rejection, loneliness and insufficient assistance from their parents and partners after giving birth.

The WHO and UNICEF emphasise the importance of community-based care, which involves providing services through the community health workforce. This approach may require collaboration with regional actors including NGOs, community organisations and youth organisations.^12^ Adolescents in South Africa face numerous obstacles in preventing unwanted pregnancies because of a lack of resources.^13^ Despite extensive efforts and legislative measures aimed at reducing AP, the incidence continues to escalate, with 33.9% of women in South Africa having experienced such pregnancies.^14^ The number of AP cases in South Africa is increasing, with a total of 90 037 teenage girls giving birth between March 2021 and April 2022 across all provinces.^15^ Given this problem, CHWs must use counselling to help reduce the prevalence of APs.

Community health workers are considered a third healthcare workforce and offer healthcare services in accordance with their training and abilities without any formal professional or paraprofessional credentials, degrees or tertiary education.^12,16^ They are not considered members of the Department of Health.^17^ The concept of a third healthcare workforce suggests that they are accountable to and responsible for the communities, NGOs and healthcare system. They are responsible for house calls, environmental sanitation, water supply, first aid treatment of minor and common ailments, dietary counselling, community development, communicable disease management, record keeping, information gathering and referrals.^16,18^ On the positive side, they offer a solid connection between the community and the public health system and often have workable ideas for enhancing both their duties and the health of the community’s residents.^19^ Community health workers can play an important role as local actors in the counselling of teenagers and promoting the physical and emotional health of the PA because of their dependability, accessibility and existing relationships with the community.

The Theory of Reasoned Action (TRA) influenced the study’s direction. The TRA was proposed in a study of American Indian adolescents to address the knowledge gap for AP and sexual behaviour.^20^ The TRA states that a person’s intentions to carry out a particular behaviour are influenced by their attitude towards that behaviour and by subjective norms. These intentions then define the behaviour that a person carries out. Individuals’ impressions of society’s standards or ideas and their motives to adhere to them are referred to as subjective norms.^20^ The TRA also includes important dimensions such as attitudes and subjective standards. As a result, CHWs must examine these constructs when conducting counselling to help AP to adopt positive behaviour and subjective standards. It is crucial that CHWs use this theory while counselling PAs to investigate the adolescents’ attitudes, feelings and subjective standards so that appropriate action may be taken to safeguard the PA and their unborn babies’ lives and to avoid difficulties. During the working and terminating phases, the TRA was used to create questions about attitudes and arbitrary standards.

According to the concise findings of Possenti et al., the counselling process should be structured into distinct stages, with therapeutic interventions carefully organised and coordinated.^11^ Furthermore, as highlighted by the European Centre for Disease Prevention and Control (ECDC), essential elements of counselling include relationship skills such as empathy, self-awareness and active listening.^11,21^ Therefore, it is essential for CHWs to be aware of their personal strengths and limitations, possess active listening skills, empathise with the emotional standpoint of PAs and effectively address their needs and desires. To assess counselling abilities, the tool was designed to guide and support CHWs through stages, incorporating insights from Possenti et al. The study aimed to evaluate the counselling skills of CHWs as they provide support to PAs in Limpopo province.

## Research methods and design

### Study design and settings

Limpopo province in South Africa is composed of five municipal districts, namely Capricorn, Mopani, Sekhukhune, Vhembe and Waterberg, which collectively have 25 local municipalities. Among these districts, only two, specifically Mopani and Vhembe, participated in the scholarship engagement study. A descriptive quantitative design was employed involving a total of 81 CHWs from these rural districts. This design allowed the researcher to collect quantitative data to objectively evaluate the counselling skills of CHWs.

### Study population and sampling strategy

The study included CHWs who were actively involved in addressing AP issues within the community. These CHWs with at least 1 year of experience, regardless of their gender or race, were eligible to participate. A simple random probability sampling method was employed to select a total of 81 CHWs from the initial pool of 90 engaged in the scholarly project. This sampling method was chosen to ensure that all respondents had an equal opportunity to participate in the study. Out of the 90 CHWs initially engaged, 81 agreed to take part, resulting in a response rate of 90%.

### Data collection

Data collection occurred over a 4-month period from July to October 2022, following a recruitment process conducted between March and June 2022. A self-administered questionnaire was utilised, consisting of demographic information and 38 closed-ended questions. These questions had dichotomous answer options (‘Yes’ or ‘No’),^22^ facilitating effective responses and data compilation for advising PAs. The questionnaire was in English and took approximately 30–60 min to complete. The questionnaire underwent peer review, during which time the abbreviation ‘SOLER’ was identified. Based on the feedback received, adjustments were made, and the abbreviation SOLER was expanded to its full form – *Sit squarely, Open posture, Lean forward, Eye contact and Relax*. Participants were provided with instructions and encouraged to seek clarification if needed.

### Data analysis

Descriptive statistics were employed using the Statistical Package for Social Scientists (SPSS, BM, New York, United States) Version 24 to analyse the data and assess the required skills for effective counselling among PAs. Cronbach’s alpha (*α*) was used to test the reliability of the questionnaire and its constructs. The reliability analysis conducted in SPSS yielded a coefficient correlation of 0.710, which is above the acceptable threshold of 0.700. Of the 38 items tested, 6 items were removed as they did not meet the minimum threshold. Table 1 provides an overview of the questionnaire’s reliability.

**TABLE 1:** Reliability statistics of the questionnaire.

Cronbach’s alpha	Cronbach’s alpha based on standardised items	Number of items (*N*)
0.710	0.735	32

### Ethical considerations

The study followed the ethical guidelines of the Helsinki Declaration and received ethical clearance from the University of South Africa, Research Ethics Committee (CREC Ref #: HSHDC/517/2016). Permission was obtained prior to data collection, and NGOs were provided with ethical information leaflets and consent letters. Participants supplied written consent, assuring informed participation. Confidentiality and anonymity were upheld, with personal identities protected and identification numbers used instead.

## Results

### Demographic profile

The study involved 81 CHWs from two rural districts in Limpopo province, with ages ranging from 20 to 79 years. The majority of the participants were from the Mopani district (63%), while the remaining (32%) were from the Vhembe district. This distribution was expected given that Mopani has more municipalities than Vhembe. The largest age group among the participants was the 30–39 age range (46%), followed by the 40–49 age range (22%). The remaining participants were divided among the 20–29 (21%), 50–59 (3.7%), 60–69 (2.5%), and 70–79 (1.2%) age ranges, indicating that the majority of the CHWs were young adults, aged 30–39 years old (46%).

Females constituted the majority of participants (87.7%), while males accounted for only 9% with 1.2% not responding. The highest educational qualification attained by participants was a degree although many respondents held a Grade 12 qualification (48.1%), followed by a high school qualification (28.4%). A smaller percentage had certificates (16.0%), diplomas (3.7%) and degrees (2.5%).

Most participants had 1–2 years of work experience (91.4%), while a minority had 3–4 years (3.7%) or 5–6 years (3.7%) of experience indicating that many participants had limited experience in the NGO field. The majority of respondents (90.1%) were CHWs, with a small percentage being administrators (3.7%), professional nurses (2.5%) or managers (2.5%). This distribution suggests that CHWs worked under the supervision of managers and professional nurses, with additional support provided by administrators. Xitsonga was the dominant language among the participants, spoken by 100% of them.

### Preparation

The results of this research reveal that preparation is extremely important for counselling sessions. Notably, the majority (99%) of the participants indicated that arranging the seating is instrumental for creating an environment to conducive effective counselling. The seating arrangements were the leading recommendation with the lowest standard deviation (SD) of 0.111. Ensuring the availability of water and tissues for clients who may respond emotionally was the next most important recommendation, with SD of 0.156. Conversely, conducting counselling in a private space with fewer distractions had a high SD of 0.218 as a highly variable recommendation. The results differed from person to person according to their opinions. Refer to Table 2 for more information.

**TABLE 2:** Preparation of descriptive statistics.

Item	Statistic					Skewness	
	*N*	Minimum	Maximum	Mean	Std. deviation	Statistic	Std. error
Conducting counselling in a private space with fewer distractions	81	1	2	1.05	0.218	4.238	0.267
Seating arrangements	81	1	2	1.01	0.111	9.000	0.267
Prepare water and tissue to provide to the client who experiences an emotional breakdown	81	1	2	1.02	0.156	6.242	0.267
Valid *N* (listwise)	81	-	-	-	-	-	-

Std., standard.

### Introduction phase

During the introduction phase, being friendly, displaying a non-judgemental attitude, maintaining appropriate eye contact, using the language that the client understands and stating the purpose of the counselling emerged (100%) as the leading recommendation with the lowest SD of 0.000. However, others (98%) mentioned that creating a relaxed atmosphere for the client to be free and comfortable was important while 96% thought that welcoming and greeting the client remains key. Using the SOLER method had the highest SD of 0.316 as a highly variable recommendation (see Table 3). The results of the introduction phase are indicated in Table 3.

**TABLE 3:** Introduction descriptive statistics.

Item	Statistic					Skewness	
	*N*	Minimum	Maximum	Mean	Std. deviation	Statistic	Std. error
Create a rapport to develop a trusting relationship	81	1	2	1.05	0.218	4.238	0.267
Welcome and greet the client	81	1	2	1.04	0.190	4.996	0.267
Use the SOLER method	81	1	2	1.11	0.316	2.522	0.267
Be friendly, and non-judgemental, and maintain an appropriate eye contact	81	1	1	1.00	0.000	-	-
Use the language that the client understands	81	1	1	1.00	0.000	-	-
Call the client by their preferred name	81	1	2	1.09	0.283	3.000	0.267
Obtain the consent	81	1	2	1.05	0.218	4.238	0.267
Assure shared confidentiality and explain the function of the multi-disciplinary team	81	1	2	1.07	0.264	3.314	0.267
Create a relaxed atmosphere for the client to be free and comfortable	81	1	2	1.02	0.156	6.242	0.267
State the purpose of counselling	81	1	1	1.00	0.000	-	-
Valid *N* (listwise)	81	-	-	-	-	-	-

Std., standard.

### Working phase

All respondents (100%) agreed that understanding how the client deals with frustrations, identifying their available support system, and recognising the challenges associated with AP play an important role in counselling. A majority (84%) of respondents suggested that giving advice during this phase should be avoided; thus counsellors should refrain from providing direct guidance. However, the leading suggestion with the lowest SD (0.000) was to ask the client about challenges related to AP. This was followed by inquiring about how clients cope with frustrations and the support systems available to them, with a SD of 0.111. The recommendation to avoid giving advice, but instead offer some suggestions had the highest SD (0.396) as a highly variable recommendation. The working phase is further indicated in Table 4.

**TABLE 4:** Working descriptive statistics.

Item	Statistic					Skewness	
	*N*	Minimum	Maximum	Mean	Std. deviation	Statistic	Std. error
Explore the feelings, challenges, and experiences	81	1	2	1.04	0.190	4.996	0.267
Ask an open-ended question ‘how do you feel about the pregnancy’	81	1	2	1.04	0.190	4.996	0.267
Be empathetic and reflect on the stated feelings	81	1	2	1.07	0.264	3.314	0.267
Apply effective communication skills i.e., paraphrasing, probing, therapeutic silence, and clanfication. You said you are ‘ok’ what do you mean, ten me more.	81	1	2	1.12	0.331	2.333	0.267
Use the appropriate tone of voice and gestures	81	1	2	1.06	0.242	3.711	0.267
Use observation skills	81	1	2	1.02	0.156	6.242	0.267
Find out how the client deals with frustration	81	1	2	1.01	0.111	9.000	0.267
Find out about the available support system	81	1	2	1.01	0.111	9.000	0.267
Ask the client about challenges related to the adolescent pregnancy	81	1	1	1.00	0.000	-	-
Ask about the various solutions to address the set challenges	81	1	2	1.06	0.242	3.711	0.267
Avoid giving advice, rather make suggestions	81	1	2	1.16	0.369	1.885	0.267
Assist the client to select the best solutions to the faced challenges	81	1	2	1.05	0.218	4.238	0.267
Find out what the client would do to prevent the reoccurrence of teenage pregnancy	81	1	2	1.02	0.156	6.242	0.267
What are your plans for the future?	79	1	2	1.09	0.286	2.952	0.271
Valid *N* (listwise)	79	-	-	-	-	-	-

Std., standard.

### Termination

According to the findings of this study, asking the client if there is a need for follow-up and stating the date, time, venue and reason for follow-up (100%) plays an important role during the termination phase. These were the leading recommendation with the lowest SD (0.000). The majority of the participants (91%) reported that the client needs to be alerted that the session is about to end to avoid terminating the session abruptly. Do not terminate the conversation abruptly, and advise the client that the session is about to come to an end had the highest SD of 0.283 as a highly variable recommendation. Refer to Table 5 for further information.

**TABLE 5:** Termination descriptive statistics.

Item	Statistic					Skewness	
	*N*	Minimum	Maximum	Mean	Std. deviation	Statistic	Std. error
Do not terminate the conversation abruptly, advise the client that the session is about to come to an end	81	1	2	1.09	0.283	3.000	0.267
Allow the client to summarise what was discussed	81	1	2	1.07	0.264	3.314	0.267
Ask the client how they feel about attending the session	81	1	2	1.01	0.111	9.000	0.267
What did the client benefit from the session?	81	1	2	1.01	0.111	9.000	0.267
Does the client need any referral and support system	80	1	2	1.02	0.157	6.202	0.269
Provide the client opportunity to ask the question	81	1	2	1.01	0.111	9.000	0.267
Ask the client if there is a need for follow-up	79	1	1	1.00	0.000	-	-
State the date, time, and venue and reason for follow up	81	1	1	1.00	0.000	-	-
Thank the client for the availability, active participation, expressing their feeling, and for coming up with solutions	81	1	2	1.07	0.264	3.314	0.267
Valid *N* (listwise)	78	-	-	-	-	-	-

Std., standard.

### Record keeping

The majority of the participants (64%) indicated that recordkeeping is not done during recording, while 36% indicated that recordkeeping is considered necessary. Record-keeping had the highest SD of (0.482) as a highly variable recommendation. The recording phase is further indicated in Table 6.

**TABLE 6:** Record keeping descriptive statistics.

Item	Statistic					Skewness	
	*N*	Minimum	Maximum	Mean	Std. deviation	Statistic	Std. error
Record-keeping	81	1	2	1.64	0.482	−0.604	0.267
Valid *N* (listwise)	81	-	-	-	-	-	-

### Correlation analysis of the constructs

Correlation is used to check the association between variables and the significance of the relationship.^22^ The result shows that the correlation is significant at the 0.01 level (two tailed) (see Table 7).

**TABLE 7:** Correlation analysis of constructs.

Item	Preparation	Introduction	Working phase	Termination	Record-keeping
**Preparation**					
Pearson correlation	-	-	-	-	-
Sig. (2-tailed)	-	-	-	-	-
*N*	81.000	-	-	-	-
**Introduction**					
Pearson correlation	0.344**	-	-	-	-
Sig. (2-tailed)	0.002	-	-	-	-
*N*	81.000	81.000	-	-	-
**Working phase**					
Pearson correlation	0.144	0.467**	-	-	-
Sig. (2-tailed)	0.206	0.000	-	-	-
*N*	79.000	79.000	79.000	-	-
**Termination**					
Pearson correlation	0.361**	0.081	0.221	-	-
Sig. (2-tailed)	0.001	0.478	0.055	-	-
*N*	78.000	78	76.000	78.000	-
**Record-keeping**					
Pearson correlation	0.040	−0.014	−0.191	−0.060	-
Sig. (2-tailed)	0.720	0.904	0.091	0.603	-
*N*	81.000	81.000	79.000	78.000	81.000

Sig., Significance.

** , Correlation is significant at the 0.01 level (2-tailed).

## Discussion

### Socio-demographic profile during counselling

Counselling skills are influenced by a CHW’s socioeconomic background. According to the findings of this study, most CHWs were matric pass, implying that they can read and write. According to Crispin et al.,^16^ their study found that age, gender, level of education and experience of CHWs all play an important role in the CHW programme. Regarding the educational attainment of CHWs, the data show that 67.7% had completed secondary school, 30.2% had finished primary education, 2.1% had attained tertiary education and a similar percentage had no formal education.^16^ Furthermore, females aged 30–50 years with low literacy levels were found to be able to conduct effective counselling skills because they are socially stable and energetic. However, educational background has no direct influence on the selection of CHWs.^17^ These members are chosen at grassroot level from the community, and the majority have a low literacy level without any formal education and are dominated by mature and married females. Given the socio-demographic findings of this study, the majority of the CHWs were females, aged 30–49 years, with some high schooling, and from areas surrounding the selected districts with limited experience. Previous research indicated that male CHWs had a 1.6 times higher likelihood of maintaining better records (odds ratio [OR] 1.64 confidence interval [CI] 1.02–2.63), while females were more likely to provide counselling (OR 0.42 CI 0.25–0.71) and empower their clients (OR 0.29 CI 0.12–0.70). These findings emphasise the importance of gender equality in effectively addressing challenges related to effective counselling and record-keeping. The health policy and system, on the other hand, emphasises that the CHW programme requires financial support to provide Integrated Community Case Management (ICCM) and Reproductive, Maternal, Newborn, Child and Adolescent Health (RMNCAH) interventions.^23^ This will enable CHWs to be knowledgeable, skilful and experienced in providing counselling services.

### Preparation during counselling

Effective counselling necessitates preparation and proper communication skills. Preparation leads to the successful application of counselling skills, and being a good listener is critical in responding effectively and asking stimulating questions that allow adolescents to be open.^11^ This implies that the counsellor must be prepared to engage in discussions and active listening to use the basic counselling techniques. This should be done in a private space to avoid distractions and to promote privacy. However, this study cautions counsellors to avoid lengthy conversing discussions, debates and rushed communication, which can lead to poor decision-making and loss of credibility. Furthermore, a collaborative and integrative approach is required to increase access to counselling services while reducing limited resources and ensuring continuity of care through referrals. Support and additional training from the primary health care system, other CHWs and NGOs are required for CHWs to successfully implement counselling skills.^24^ Refresher training and capacity-building workshops should be implemented to address knowledge gaps and promote effective counsellors.^25^ This is required for CHWs to be change agents. The demographic profile revealed that the majority of CHWs lacked the necessary qualifications and experience.

### Introduction phase during counselling

According to Possenti et al.,^11^ this study highlights the significance of self-awareness in understanding the factors that can hinder or support relationship building. Effective counselling skills rely on self-awareness, empathy and active listening. Building rapport and empathy are crucial for facilitating effective communication.^25^ A study conducted in Nigeria suggests that patience and respect should be exercised, and the use of technical language should be avoided to enhance communication skills.^26^ Creating a welcoming environment, ensuring consent and confidentiality^27^ and addressing record-keeping are essential for meeting the needs of PAs. Possenti et al.’s findings influenced the categorisation of counselling skills into phases, emphasising the importance of conducting the counselling process accordingly.^11^ The counselling process consists of phases such as initial greeting, relationship building, problem solving, goal setting, solution proposal, summarising, evaluation, termination and/or referral, closure and final greeting, with no specific mention of documentation and record keeping. SOLER method promotes non-verbal listening in effective communication.^28^ World Health Organization and UNICEF stress the importance of training CHWs to offer counselling and information on topics like unsafe abortion prevention, family planning, high-risk pregnancies, parenting, mental health and psychological support referrals.^12^

### Working phase during counselling

The TRA was used to formulate attitude questions, allowing CHWs to explore adolescents’ feelings, challenges and attitudes towards AP. This helped understand the behaviours affecting pregnancy and address frustrations and challenges effectively. Adolescent pregnancy raises global concerns because of the risks of repeated pregnancy, health impact and socioeconomic challenges.^27^ Pregnant adolescents are considered vulnerable and psychologically immature.^29^ The study highlights the importance of offering assistance and support to PAs. Addressing their challenges necessitates offering care, support and counselling to help them navigate obstacles responsibly and knowledgeably, Possenti et al.^11^ emphasise the importance of counsellors employing effective communication skills during this phase. Successful counselling for PAs involves elements such as problem assessment, exploring alternatives, setting achievable goals, fostering collaboration and establishing supportive networks. Tailoring counselling to individual needs and avoiding giving direct advice are also important considerations.^25^

### Termination and counselling

According to Possenti et al.,^11^ to achieve a successful conclusion of counselling sessions, key factors such as summarising, evaluating, referring, strengthening teamwork, networking, closing and final greetings should be considered. Avoiding rushing is essential. The TRA can bridge knowledge gaps on sexual behaviours and AP, as suggested by a previous study among American Indian adolescents.^20^ The TRA guided CHWs in pregnancy counselling to promote positive behaviours and informed decision-making amid challenges. Gradual termination, summarisation, evaluation, referral, continuity of care, closure and client acknowledgements were supported. Collaborative termination in the final session was recommended for successful termination and client empowerment in decision-making.^30^

### Record keeping

According to the findings of this study, no records were kept. In a Kenyan study, education, age, gender and experience were identified as factors impacting record keeping, with males demonstrated 1.6 times better skills compared to females (OR 1.64 CI 1.02–2.63).^16^ The study also found that inadequate record-keeping was associated with the respondents’ educational background, gender and experience. Significant correlations between age and positive record keeping (*p* = 0.0001) proper utilisation of job aids (*p* = 0.0001), client satisfaction (*p* = 0.018) and client enablement (*p* = 0.001) were observed.^16^ Specifically, the respondents were primarily female, aged between 30 and 39 years with Grades 10 and 12 education levels. Charurvedi et al.^31^ acknowledge a knowledge gap in history taking during counselling, attributed to ignorance. Conversely, Myers et al.^24^ found that CHWs need substantial support to handle their workload and address counselling-related challenges. The analysis of socio-demographic characteristics provided insights into the study participants. A correlation was found between record keeping and CHWs’ socio-demographic profile, aiding in identifying their specific counselling needs and planning relevant workshops. Health policy and system evidence highlight the importance of training CHWs in counselling skills, stigma reduction, behaviour change and community engagement for effective CHW programmes.^23^

### Implications of findings for evaluation of community health workers counselling skills

To enhance the counselling skills and overall performance of CHWs, it is crucial to offer regular workshops and training. It is important to address the lower proficiency of female CHWs in record keeping ensuring counsellors are well informed and can provide relevant information. Teaching relational abilities such as empathy, self-awareness and active listening is vital for effective counselling sessions. Adopting a collaborative approach is necessary to foster networking, provide continuous support and ensure ongoing treatment.

### Strengths and limitations

This study focussed on CHWs employed by NGOs in rural districts of Limpopo. The research is valuable for local communities, healthcare providers, NGOs and policymakers in similar settings, providing insights on enhancing CHWs’ counselling skills. Due to the focus of the study on CHWs in rural districts of Limpopo, the findings are context specific and may not be generalised to other settings.

## Conclusion

Counselling plays a crucial role in positively impacting the lives of PAs and their unborn children. The study findings revealed several important aspects related to counselling practices. Conducting counselling in a private space with minimal distractions during preparation showed considerable variability as a recommendation, with a high SD of 0.218. Similarly, avoiding advice-giving and instead offering suggestions had the highest SD of 0.396, indicating significant variability as a recommendation. The use of the SOLER method had the highest SD of 0.316, indicating considerable variability as a recommendation. The study highlighted a prevalent issue of inadequate recordkeeping during counselling sessions. A significant majority (64%) of participants reported that recordkeeping is not practised, while the remaining 36% acknowledged the importance of recordkeeping during counselling sessions. Community health workers must be trained in counselling skills to provide support and adequate information while also alleviating the burden of counselling in the education and health systems. Emphasising the importance of record-keeping in counselling is crucial to accurately document completed sessions, challenges faced, interventions implemented, referrals made and the need for follow-up. Community health workers should at least be able to read and write for proper record keeping. The inclusion of young counsellors is paramount. This will encourage PAs to be open and to talk freely to their peers in the language they understand. They can bridge any linguistic or cultural barriers that may exist, ensuring effective communication. Promoting the involvement of male partners in counselling is important to offer emotional support to PAs and help them navigate the challenges and uncertainties associated with pregnancy.
